# Specific alterations of gut microbiota in diabetic microvascular complications: A systematic review and meta-analysis

**DOI:** 10.3389/fendo.2022.1053900

**Published:** 2022-12-05

**Authors:** Jinni Hong, Tingting Fu, Weizhen Liu, Yu Du, Cunyun Min, Datao Lin

**Affiliations:** ^1^ Department of Traditional Chinese Medicine, Guangdong Provincial People’s Hospital, Guangdong Academy of Medical Sciences, Guangzhou, China; ^2^ Department of Traditional Chinese Medicine Guangdong Provincial Institute of Geriatric, Guangzhou, China; ^3^ Department of Parasitology, Zhongshan School of Medicine, Sun Yat-sen University, Guangzhou, China

**Keywords:** diabetic kidney disease (DKD), diabetic peripheral neuropathy (DPN), diabetic retinopathy (DR), gut microbiota (GM), diabetic microvascular complication

## Abstract

**Background:**

The role of gut microbiota in diabetes mellitus (DM) and its complications has been widely accepted. However, the alternation of gut microbiota in diabetic microvascular complications (DC) remains to be determined.

**Methods:**

Publications (till August 20^th^, 2022) on gut microbiota in patients with DC were retrieved from PubMed, Web of Science, Embase and Cochrane. Review Manager 5.3 was performed to estimate the standardized mean difference (SMD) and 95% confidence interval (CI) and calculate alpha diversity indices and the relative abundance of gut microbiota between patients in DC v.s. DM and DC v.s. healthy controls (HC).

**Results:**

We included 13 studies assessing 329 patients with DC, 232 DM patients without DC, and 241 HC. Compared to DM, patients with DC shared a significantly lower Simpson index (SMD = -0.59, 95% CI [-0.82, -0.36], p < 0.00001), but a higher ACE index (SMD = 0.42, 95% CI[0.11, 0.74], p = 0.009). Compared to HC, DC patients held a lower ACE index (SMD = -0.61, 95% CI[-1.20, -0.02], p = 0.04). The relative abundances of phylum Proteobacteria (SMD = 0.03, 95% CI[0.01, 0.04], p = 0.003, v.s. HC) and genus *Klebsiella* (SMD = 0.00, 95% CI[0.00, 0.00], p < 0.00001, v.s. HC) were enriched, accompanying with depleted abundances of phylum Firmicutes (SMD = -0.06, 95% CI[-0.11, -0.01], p = 0.02, v.s. HC), genera *Bifidobacterium* (SMD = -0.01, 95% CI[-0.02,-0.01], p < 0.0001, v.s. DM), *Faecalibacterium* (SMD = -0.01, 95% CI[-0.02, -0.00], p = 0.009, v.s. DM; SMD = -0.02, 95% CI[-0.02, -0.01], p < 0.00001, v.s. HC) and *Lactobacillus* (SMD = 0.00, 95% CI[-0.00, -0.00], p < 0.00001, v.s. HC) in DC.

**Conclusions:**

Gut microbiota perturbations with the depletion of alpha diversity and certain short-chain fatty acids (SCFAs)-producing bacteria were associated with the pathology of DC. Therefore, gut microbiota might serve as a promising approach for the diagnosis and treatment of DC. Further investigations are required to study the mechanisms by which gut dysbiosis acts on the onset and progression of DC.

## Introduction

Diabetes mellitus (DM) is an epidemic and accounts for 80% of premature deaths globally (1, 2). As of 2021, there are approximately 537 million DM patients in the world, with an extended increment to 700 million by 2045 (3). DM is hardly a disease with mere elevation in blood glucose, it brings along a plethora of microvascular complications including diabetic retinopathy (DR), diabetic kidney disease (DKD) and diabetic peripheral neuropathy (DPN) in most cases. These microvascular complications are responsible for the high mortality and morbidity rate in DM patients and account for marked social and economic burdens (4). Although numerous treatments for DM and resulting complications were available, cases are still on the rise. DR is the leading cause of blindness ophthalmic disorder in the population on working age, with a prevalence of 77.8% in 15 years of DM patients (5–7). DKD occurs in approximately 40% of DM, and 30%-90% of DM might suffer from DPN (8). These trends highlight the urgency for a better understanding of diabetic microvascular complications (DC) (9).

The rapid scientific interest in gut microbiota coincided with the global increase in DM (9). The advent of next-generation sequencing technology has greatly enhanced our understanding of gut microbiota and host health (10, 11). The gut microbiota was a complex microbial community, represented by 1500 different species (12, 13). DM and its complications have been linked with dysbiosis of the gut microbiota (14). Differences in gut microbiota composition have been observed in animal models as well as patients with DM and complications such as DKD, DR and DPN. In particular, perturbed Bacteroidetes/Firmicutes eubiosis was proven to be associated with increased intestinal permeability, with bacteria byproducts infiltrated through a leaky gut barrier, triggering inflammatory responses of diabetes. Species *Lactobacillus fermentum*, *Akkermansia muciniphila, Bacteroides fragilis* and *Roseburia intestinalis* were demonstrated to be linked to insulin sensitivity and glucose metabolism. Phyla Bacteroidetes, Actinobacteria and Mucoromycota were depleted, while genera *Acidaminococcus*, *Escherichia* and *Enterobacter* were enriched in patients with DR compared to HC (15). In DPN, the richness of Firmicutes and Actinobacteria was elevated, while Bacteroidetes was depleted. At the genus level, *Bacteroides* and *Faecalibacterium* were significantly decreased, whereas *Lachnoclostridium* and *Ruminococcus* were enriched (16). In DKD, there was a marked increase in genera *Acidaminococcus, Selenomonadales*, *Bilophila* and *Shigella*, as well as phylum Proteobacteria, and the richness of species *Syntrophaceticus schinkii* and *Citrobacter farmeri* was positively correlated with the urinary albumin creatinine ratio (17). Notably, *Akkermansia* in the gut may contribute to the effect of metformin, the most commonly prescribed drug for DM, on glucose metabolism (18, 19).

Although a plethora of studies had characterized the gut microbiota of DC with promising findings, the relationship between them was still controversial. A meta-analysis on more than 2000 studies on microbiota suggested the alternation of microbiota to differentiate healthy and diseased populations and serve as bio-markers for the diagnosis or treatment of DM (20). Therefore, we performed a meta-analysis of gut microbiota from patients with DC and explored the diversity and bacterial characteristics of the gut microbiota in DC.

## Methods

The scheme of the review was registered in PROSPERO with registration number CRD42022353144. We followed the Preferred Reporting Items for the Systematic Reviews and Meta-analyses (PRISMA) reporting guidelines (21).

### Retrieval strategy

We searched PubMed, Web of Science, Embase and Cochrane databases for observational case-control studies or cross-sectional studies from inception to August 20^th^, 2022, with the search strategy: ((diabetic retinopathy) OR (diabetic microvascular complications) OR (diabetic peripheral neuropathy) OR (diabetic neuropathy) OR (diabetic kidney disease) OR (diabetic nephropathy)) AND (Microbiota OR Microbiome). This online strategy was augmented by a bibliographic search to identify other potentially eligible publications. Records were restricted to studies published in English and conducted on humans. All duplicated records were removed.

### Selection criteria

Two reviewers (JH and TF) independently screened titles, abstracts, and full-text articles for inclusion and resolved differences through consensus. The eligible criteria included studies reporting DM patients with DC, setting healthy controls (HC) or DM patients without DC as controls, and performing gut microbiota analysis and reporting alpha diversity or abundance measures as outcome results. Records in review form or without full-text available were excluded.

### Data extraction, quality assessment and risk of bias assessment

Using standardized forms, two reviewers (JH and TF) extracted information on the general study and participant characteristics, as well as the outcome results including alpha diversity indices and relative abundance of microbiota at the phylum and genus level. Alpha diversity referred to species richness and evenness within communities or habitats, including observed number of operational taxonomic units (OTUs), Chao1, ACE, Simpson and Shannon indices, which were usually plotted using the R package. Specially, OTUs and Chao1 reflected the number of species in the community, regardless of the abundance of each species in the community. ACE considered a wider range of rare species, and adjusted the coefficient of variation and sample coverage to make data more reasonable. The Simpson and Shannon indices reflected the diversity of the bacterial communities, which were affected by the species richness and evenness in the community. Mean and standard deviation (SD) were collected from text or tables in articles, and results presented in graphs were abstracted using GetData Graph Digitizer v.2.22 (Australia) software. Since a large part of data on microbiota was presented as a box-plot, when the mean and SD were not available, the median and quartile range were extracted and estimated by the calculator of Review Manager 5.3. A third reviewer (DL) confirmed the abstracted data.

Newcastle-Ottawa Quality Assessment Scale (NOS) was used to evaluate the literature quality (22), and a score ≥ 5 indicated adequate quality for inclusion in the present review (23). A funnel plot was constructed to assess publication bias.

### Statistical analysis

Data were exported to Review Manager 5.3 (Nordic Cochrane Centre, Copenhagen, Denmark) software for statistical meta-analysis. We pooled the mean difference (MD) or standardized mean difference (SMD) for continuous outcomes, and a 95% confidence interval (CI) was utilized to estimate the prediction. The heterogeneity was evaluated by I^2^ statistic, and a fixed and random effect model was performed when I^2^ < 50% and I^2^ > 50%, respectively. Two-sided p values were statistically significant at less than 0.05.

## Results

### Literature screening results

**Figure 1:**
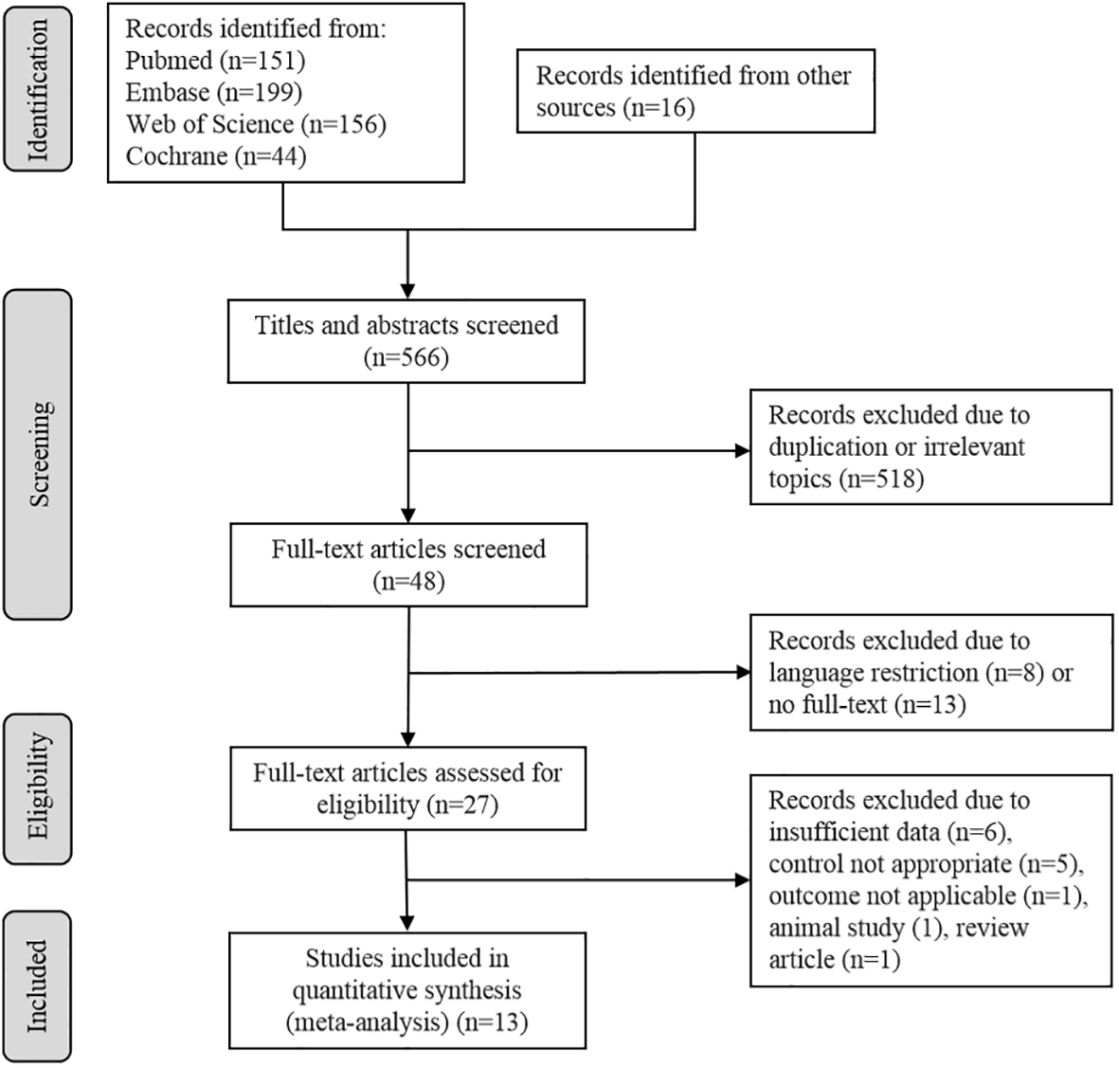
Flow diagram describes the selection process of the included studies.

A total of 566 potentially eligible articles were retrieved, including 16 records identified from other sources. By screening abstracts and titles, 518 articles were excluded for duplication or irrelevant topics, leaving 48 studies. After that, eight articles were abandoned for language restriction and 13 were due to no full-text. Then 27 full -text articles were screened for eligibility. The most common reason for exclusion was insufficient data (n = 6), five records were excluded because the control was not appropriate, one was an animal study, one was a review article, and one did not report data in applicable form, leaving 13 studies meet all the inclusion criteria (**Figure 1**).

### Characteristics of the included studies

The total 13 studies (15–17, 24–33) captured 329 patients with DC, 232 DM patients without DC, and 241 HC, with the territorial scope covering India (2/13), USA (2/13) and China (9/13). Seven (7/13) of the studies focused on DKD, five (5/13) focused on DR, and one (1/13) focused on DPN. The methodology of composition analysis also varied widely, with 16S rRNA gene sequencing being the most common (6/13), followed by 16S rDNA-based high-throughput sequencing (4/13) and three (3/13) with metagenomic sequencing. The basic characteristics of the articles included in the study were shown in **Table 1**.

**Table 1:** Characteristics of the included studies.

Study	Author	Year	Region	Disease	DC		DM		HC		Analysis methods
					N, M/F	Age(year)	N, M/F	Age(year)	N, M/F	Age(year)
1	L. Zhang	2022	Shandong, China	DKD	12,6/6	61.67 ± 8.75	12,7/5	57.08 ± 8.59	14,7/7	58.86 ± 7.36	Metagenomic sequencing
2	R. Chen	2022	Guangdong, China	DKD	22,11/11	60	–	–	22,11/11	57	16S rDNA
3	X. He	2022	Shanxi, China	DKD	10,9/1	56.00 ± 14.97	10,8/2	64.90 ± 7.37	–	–	Metagenomic sequencing
4	Z. Zhou	2021	Chongqing, China	DR	21,14/7	59.57 ± 9.09	14,8/6	61.93 ± 6.20	15,7/8	56.13 ± 8.88	16S rDNA
5	P. Ye	2021	Zhejiang, China	DR	45,25/20	59.9 ± 11.3	90,50/40	60.9 ± 9.9	–	–	16S rRNA
6	T. Das	2021	Hyderabad, India	DR	28,21/7	55.07	25,14/11	57.3	30,17/13	52.2	16S rRNA
7	X. Du	2021	Tianjin, China	DKD	43,32/11	60.86 ± 5.69	–	–	37,25/12	61.78 ± 6.40	16S rDNA
8	Y. Huang	2021	Hunan, China	DR	25,15/10	60.28 ± 10.5	25,11/14	62.52 ± 7.58	25,9/16	57.80 ± 10.06	16S rRNA
9	Y. Wang	2020	Nanjing, China	DPN	45,25/20	58.55 ± 6.61	21,12/9	59.33 ± 10.21	14,8/6	58.06 ± 6.39	16S rDNA
10	R. Jayasudha	2020	Hyderabad, India	DR	24,18/6	54.5	21,13/8	57.5	30,17/13	52.2	Metagenomic sequencing
11	S. Tao	2019	Sichuan, China	DKD	14,9/5	52.93 ± 9.98	14,9/5	53.29 ± 9.00	14,9/5	52.86 ± 9.91	16S rRNA
12	M. Salguero	2019	Amarillo, TX, USA	DKD	20,9/11	62.8 ± 3.6	–	–	20,11/9	58.5 ± 4.1	16S rRNA
13	M. Al-Obaide	2017	Amarillo, TX, USA	DKD	20	64.4 ± 2.3	–	–	20	54.3 ± 3.2	16S rRNA

DC, Diabetic Microvascular Complication; DM, Diabetes Mellitus; HC, Healthy Control; N, Number; M/F, Male/Female; DKD, Diabetic Kidney Disease; DR, Diabetic Retinopathy; DPN, Diabetic Peripheral Neuropathy.

### Quality of included studies

The NOS scores showed one (1/13) study with a score of six, ten (10/13) with a score of seven, and two (2/13) with a score of eight, indicating a relatively high quality of the studies selected (**Supplementary Table S1**).

### Alpha diversity between DC and DM

A total of ten trials were obtained to assess alpha diversity, including 279 DC patients, 222 DM patients and 201 HC. Five indices were obtained including estimates of richness (OTUs, ACE and Chao1), and diversity (Simpson and Shannon).

**Figure 2:**
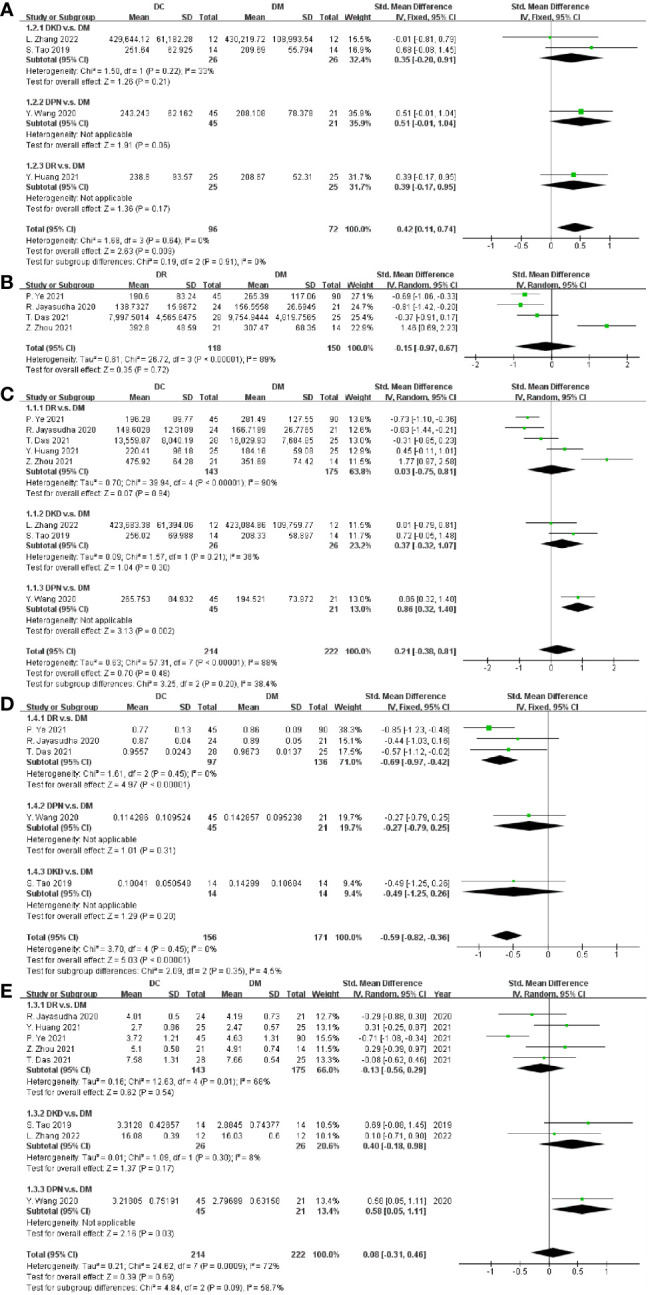
Forest plots of alpha diversity in DC v.s. DM. **(A)** ACE; **(B)** OTUs; **(C)** Chao1; **(D)** Simpson; **(E)** Shannon.

Regarding richness, four studies provided data on ACE, and the pooled estimate showed a significant increase in ACE in DC (SMD = 0.42, 95% CI[0.11, 0.74], p = 0.009) in **Figure 2A**. Four and eight studies reported the OTUs and Chao1 index in DC v.s. DM respectively, with non-significant differences between groups (OTUs SMD = -0.15, 95% CI[-0.97, 0.67], p = 0.72, I^2^ = 89%; Chao1 SMD = 0. 21, 95% CI[-0.38, 0.81], p = 0.48, I^2^ = 88%) in **Figures 2B, C**. To explore the potential sources of the existed heterogeneity in OTUs, subgroup analysis on the nation and city was done in **Supplementary Figures S1A, B**. The heterogeneity decreased to 10% in nation except China, and further analysis revealed that city bias might be the source of heterogeneity. To explore the potential sources of the heterogeneity in Chao1, subgroup analysis on DC including DR, DPN and DKD was performed. Since the heterogeneity still existed, subgroup analysis on the nation was done, and results showed the heterogeneity vanished in the nation except China (**Supplementary Figure S1C**).

Regarding diversity, five studies provided data on DC, and the pooled estimate showed a significant decrease of the Simpson index in DC (SMD = -0.59, 95% CI [-0.82, -0.36], p < 0.00001), as shown in **Figure 2D**. Eight studies measured and reported the Shannon index in DC and DM, but our meta-analysis revealed no significant difference between them (SMD = 0.08, 95% CI [-0.31 0.46], p = 0.69), as depicted in **Figure 2E**. The type of DC and nation bias might be the sources of high heterogeneity (I^2^ = 72%), as depicted in **Figure 2E** and **Supplementary Figure S1D**.

Since only one study on DPN (16) was included in this study, to explore the source of heterogeneity caused by it, we performed a meta-analysis on studies excluding DPN and compared the results with meta-analysis on studies including DPN. As depicted in **Supplementary Figure S2**, a meta-analysis of studies excluding DPN was conducted. There was a significant increase in the Simpson index (SMD = -0.67, 95% CI [-0.93, -0.41], p < 0.00001), with no significant differences found in the Shannon index (SMD = -0.01, 95% CI [-0.39, 0.38], p = 0.97), ACE (SMD = 0.37, 95% CI[-0.02, 0.76], p = 0.06), and Chao1 (SMD = 0.11, 95% CI[-0.51, 0.74], p = 0.72) between DC and DM. Subgroup analysis on the type of DC and nation was performed in **Supplementary Figure S3**, and similar conclusions with studies including DPN were drawn that the type of DC and nation bias might be the sources of heterogeneity in the Shannon index and Chao1, respectively. Above all, the tendency of the results with or without DPN was consistent.

### Alpha diversity between DC and HC

**Figure 3:**
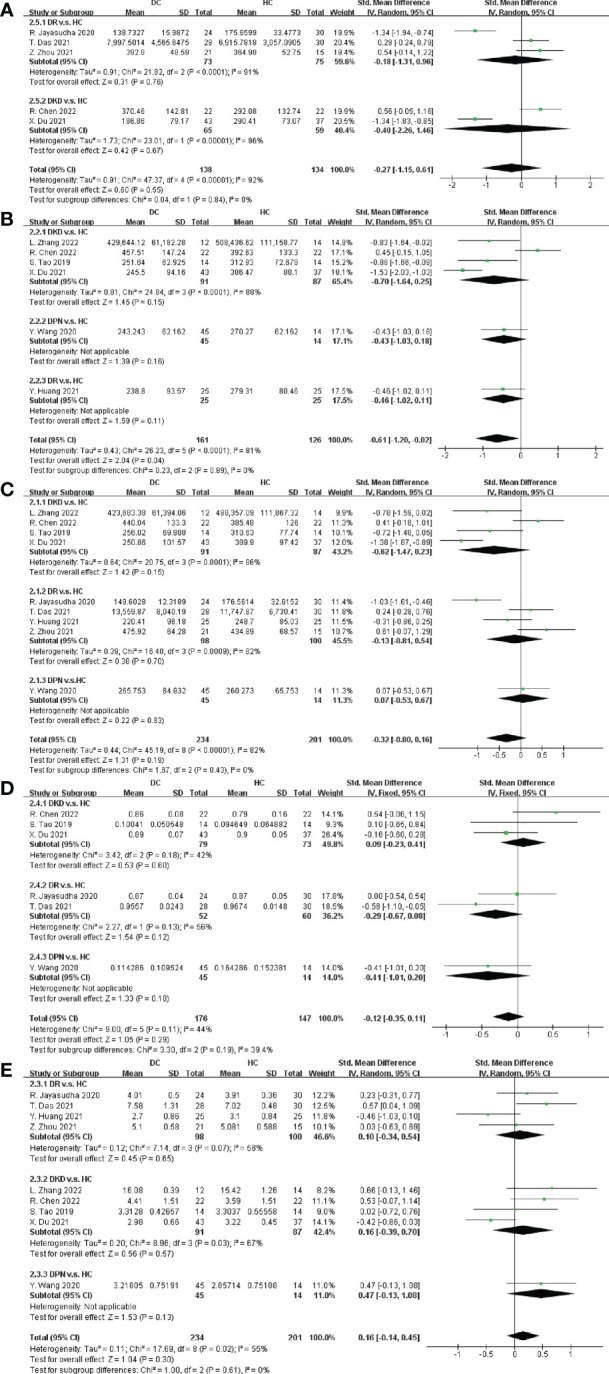
Forest plots of alpha diversity in DC v.s. HC. **(A)** OTUs; **(B)** ACE; **(C)** Chao1; **(D)** Simpson; **(E)** Shannon.

Furthermore, alpha diversity between DC and HC was compared in terms of OTUs, ACE, Chao1, Simpson and Shannon. Five studies reported a relative abundance of bacterial taxa in terms of OTUs, while a non-significant difference was found between DC and HC (SMD = -0.27, 95% CI[-1.15, 0.61], p = 0.55), as depicted in **Figure 3A**. Since there was a high heterogeneity (I^2^ = 92%), subgroup analysis on the type of DC and nation was performed. Nevertheless, the heterogeneity still existed in either subgroup of DC and nation, and the funnel plots were symmetric overall as shown in **Supplementary Figure S4**.

In **Figure 3B**, from the six trials included in this meta-analysis, we obtained that the SMD of ACE was -0.70 in DKD (95% CI: -1.64, 0.25), -0.43 in DPN (95% CI: -1.03, 0.18) and -0.46 in DR (95% CI: -1.02, 0.11), with no significant difference. Nevertheless, the overall SMD of DC was significantly decreased (SMD = -0.61, 95% CI[-1.20, -0.02], p = 0.04). Nine studies provided Chao1 data in DC v.s. HC, with a non-significant difference estimated after meta-analysis (SMD = -0.32, 95% CI[-0.80, 0.16], p = 0.19), as depicted in **Figure 3C**.

Regarding community diversity, **Figures 3D, E** showed that six and nine trails provided Simpson and Shannon data respectively. However, the differences in community diversity between DC and HC estimated by Simpson and Shannon were not significant, with Simpson SMD = -0.12, 95% CI[-0.35, 0.11], p = 0.29, and Shannon SMD = 0.16, 95% CI[-0.14, 0.45], p = 0.30. Nation bias assessments and funnel plots for the alpha diversity were shown in **Supplementary Figure S5**.

Since only one study on DPN was included in this meta-analysis, considering the representativeness and potential bias, a meta-analysis of studies excluding DPN was performed in **Supplementary Figure S6**. Compared with the meta-analysis of studies including DPN, similar results were found in ACE (SMD = -0.65, 95% CI[-1.36, 0.07], p = 0.08), Chao1 (SMD = -0.37, 95% CI[-0.90, 0.16], p = 0.17), the Simpson index (SMD = -0.07, 95% CI[-0.32, 0.17], p = 0.55), and the Shannon index (SMD = 0.12, 95% CI[-0.20, 0.44], p = 0.47) between DC and HC. Nation bias assessments and funnel plots of alpha diversity were shown in **Supplementary Figure S7**.

### Different abundance of microbiota at the phylum level

**Figure 4:**
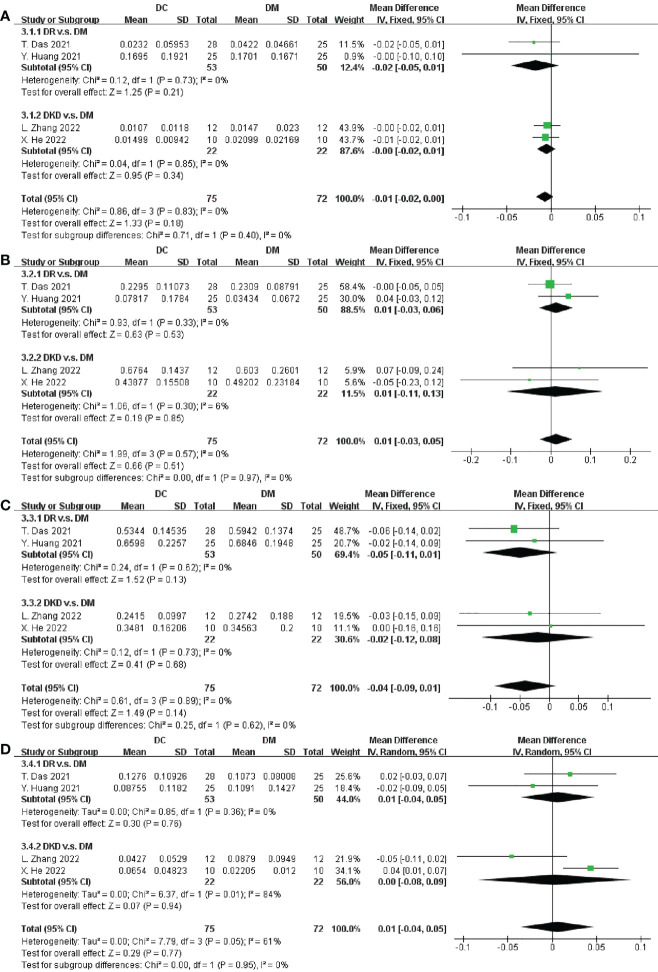
Forest plots of microbiota at the phylum level in DC v.s. DM. **(A)** Actinobacteria; **(B)** Bacteroidetes; **(C)** Firmicutes; **(D)** Proteobacteria.

Five studies involving 187 participants described the distinct taxa at the phylum level. Examining taxonomic distribution at the phylum level did not reveal remarkable differences between DC and DM in Actinobacteria, Bacteroidetes, Firmicutes, Proteobacteria, Fusobacteria, and Verrucomicrobia (**Figure 4** and **Supplementary Figure S8**).

**Figure 5:**
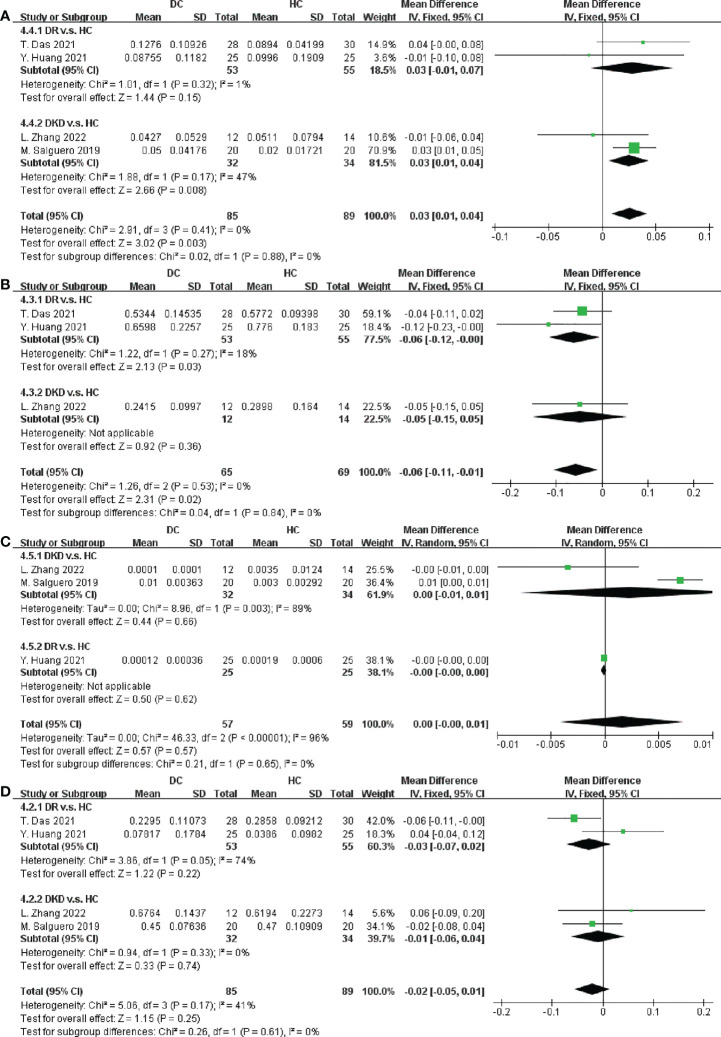
Forest plots of microbiota at the phylum level in DC v.s. HC. **(A)** Proteobacteria; **(B)** Firmicutes; **(C)** Fusobacteria; **(D)** Bacteroidetes.

We next performed an analysis of microbiota at the phylum level between DC and HC. Among the most abundant species, we observed that DC patients were enriched in Proteobacteria (SMD = 0.03, 95% CI[0.01, 0.04], p = 0.003), and depleted in Firmicutes (SMD = -0.06, 95% CI[-0.11, -0.01], p = 0.02), in **Figures 5A, B**. Specially, the abundance of Proteobacteria inDKD was significantly higher than that in HC (SMD = 0.03, 95% CI[0.01, 0.04], p = 0.008) and Firmicutes was significantly depleted in DR when compared to HC (SMD = -0.06, 95% CI[-0.12, -0.00], p = 0.03).

Furthermore, data in **Figures 5C, D** and **Supplementary Figure S9** demonstrated there were no significant differences in the relative abundance of Fusobacteria, Bacteroidetes, Actinobacteria and Verrucomicrobia between DC and HC.

### Different abundance of microbiota at the genus level

**Figure 6:**
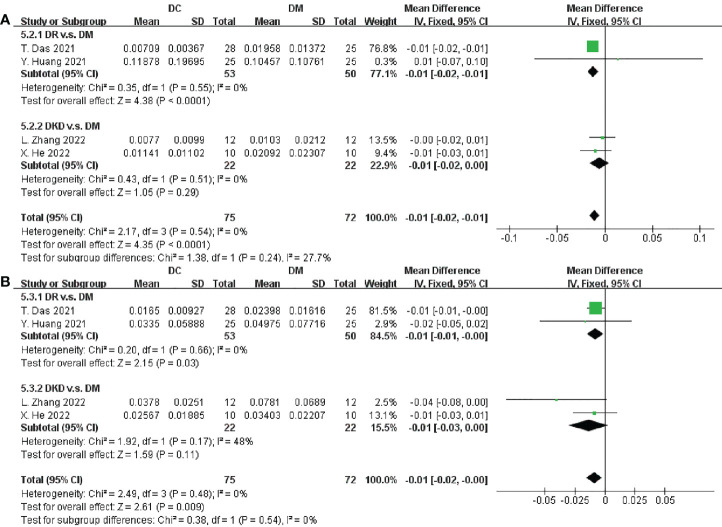
Forest plots of microbiota at the genus level in DC v.s. DM. **(A)** *Bifidobacterium*; **(B)** *Faecalibacterium*.

From the 11 trials included in this meta-analysis, we obtained that there was a significantly deplete in richness of *Bifidobacterium* and *Faecalibacterium* in DC when compared to DM (SMD = -0.01, 95% CI[-0.02,-0.01], p < 0.0001; SMD = -0.01, 95% CI[-0.02, -0.00], p = 0.009, respectively), as depicted in **Figures 6A, B**. However, the abundances of *Alistipes*, *Prevotella*, *Ruminococcus*, *Lachnospira*, *Roseburia*, *Clostridium, Blautia, Escherichia*, *Eubacterium*, *Parabacteroides*, *Mitsuokella* and *Lactobacillus* varied, and we did not find any relatively consistent results after meta-analysis (**Supplementary Figures S10 and 11**).

**Figure 7:**
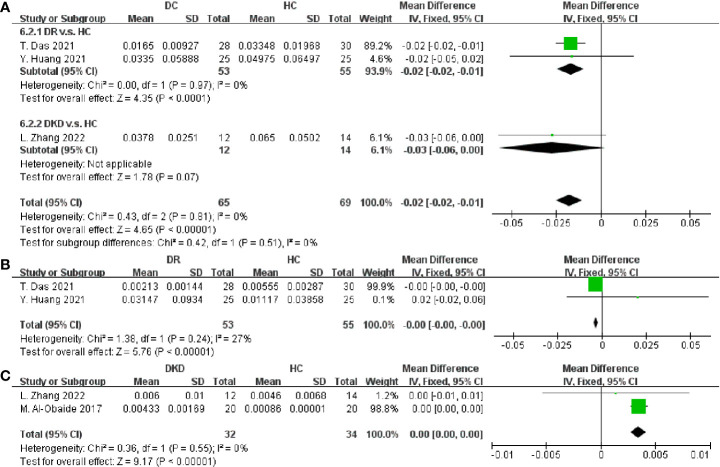
Forest plots of microbiota at the genus level in DC v.s. HC. **(A)** *Faecalibacterium*; **(B)** *Lactobacillus*; **(C)** *Klebsiella*.

**Figure 8:**
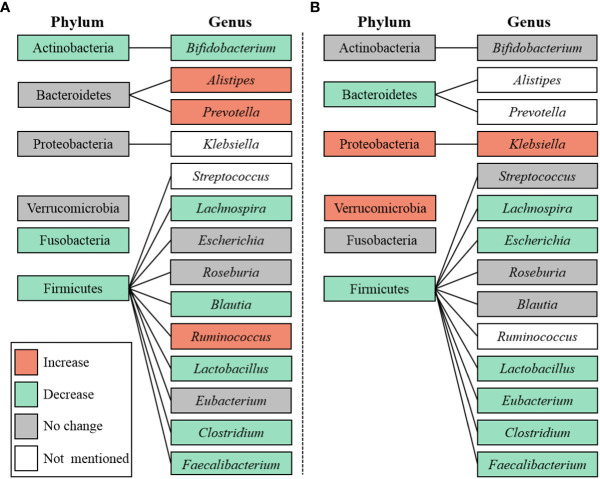
Microbiota at the phylum and genus level. **(A)** DC v.s. DM; **(B)** DC v.s. HC.

After that, the taxa between DC and HC were compared in **Figure 7**. *Faecalibacterium* and *Lactobacillus* were proven to be decreased in DC (SMD = -0.02, 95% CI[-0.02, -0.01], p < 0.00001; SMD = -0.00, 95% CI[-0.00, -0.00], p < 0.00001; respectively), as well as an increase of *Klebsiella* (SMD = 0.00, 95% CI[0.00, 0.00], p < 0.00001). Besides, no consistent results were found in the relative abundances of *Streptococcus*, *Roseburia*, *Clostridium*, *Blautia*, *Escherichia*, *Eubacterium*, *Bifidobacterium* and *Lachnospira* (**Supplementary Figures S12 and S13**). In this study, we summarized the alternation tendency of gut microbiota in DC compared to DM and HC respectively in **Figure 8**.

## Discussion

To our knowledge, this meta-analysis was the first to assess alpha diversity and microbiota perturbations in DC compared with DM and HC. This study yielded three major insights into the gut microbial changes in DC. Firstly, patients with DC exhibited a significantly higher richness but lower diversity in gut microbiota when compared to DM. And when compared to HC, DC held a lower richness in gut microbiota. Secondly, the abundance of phylum Proteobacteria was enriched and Firmicutes was depleted in DC when compared to HC. Last, the abundances of genera *Bifidobacterium* and *Faecalibacterium* were depleted in DC when compared to DM. And when compared to HC, DC exhibited lower abundances of *Faecalibacterium* and *Lactobacillus* but a higher abundance of *Klebsiella*. All these changes may be associated with the pathology of DC, and serve as promising targets for the management of DC.

One common indicator of dysbiosis is a modified overall microbial alpha diversity, which denotes the relative abundance of microbial species in space and time within a specific community. Alpha diversity indices include OTUs, ACE, Chao1, Simpson and Shannon. ACE and Chao1 focus on species richness. Simpson and Shannon strengthen diversity. Generally, lower alpha diversity was observed in obesity and diabetes, and was considered detrimental to the host (34). Alpha diversity in richness and diversity declined in DR and DKD when compared to HC (28, 29). In contrast, no difference was found in gut microbiota between DM patients with or without cognitive impairment (35). DPN resulted in a more severe disruption of microbiota community richness than DM (16). These findings indicated that the profile of gut microbiota was altered in patients with DC, but further exploration was needed due to the extremely inconsistent findings.

Phylum Proteobacteria was gram-negative bacteria and proved to be significantly different in gut microbiota between DM patients with or without gastrointestinal autonomic neuropathy (36). Proteobacteria was proven to be negatively related to human health and found with a higher abundance in the obese population than the non-obese population (34, 37). The mechanism might be related to the regulation of bile acids, a metabolic controller (38). In line with these results, Proteobacteria was found significantly enriched in DC when compared to HC in this study, concluding that with the risk of DC increased, the abundance of Proteobacteria increased. These findings may further reveal that the alternation of gut microbiota at the phylum level is closely related to DC.

The phyla Firmicutes and Bacteroidetes accounted for more than 90% of the total community of human gut microbiota. Alterations affecting Firmicutes and Bacteroidetes were first described in obese subjects who exhibited increased abundances of Firmicutes at the expense of Bacteroidetes (39). Firmicutes was positively linked with inflammation and the modulation of metabolism, and was supposed to be more efficient at calorie absorption and weight gain (40–42). Bacteroidota was significantly reduced in DR patients, and Firmicutes to Bacteroidetes ratio was proven elevated in gestational diabetes patients (15). However, in opposition to these results, no change or even decreased Firmicutes to Bacteroidetes ratio in obese subjects was reported (43–45). In our study, Firmicutes were significantly depleted in DC compared to HC. The reason might be linked to less bacterial diversity in obese patients than the non-obese subjects (46). Therefore, further research involving this parameter should be high on the list.

In line with the results presented in this study concerning the significant decrease of genera *Bifidobacterium*, *Lactobacillus* and *Faecalibacterium* in DC (p < 0.05), similar results were observed in DR in Zhou’s study (31). *Bifidobacteria* and *Lactobacillus* were gram-positive microorganisms, which participated in the restoration of the intestinal mucosal barrier (47, 48). Both of them were inversely associated with inflammation, hyperglycemia, and insulin resistance (49–51). *Bifidobacteria* was demonstrated to produce bacteriocin that blocks adherence to the mucosa and preserves gut barrier function (52). DM patients presented a significantly lower richness of *Bifidobacterium* compared to healthy subjects (53, 54). Probiotics, as well as yogurt or milk containing *Bifidobacterium* and *Lactobacillus*, decreased fasting blood glucose and glycosylated hemoglobin type A1c (HbA1c) in DM patients (55, 56). Drawing a conclusion that *Bifidobacteria* and *Lactobacillus* decreased as the risk of DC increased. The mechanism might be linked to the depletion of potential intestinal pathogens, enhancement of intestinal antioxidant ability and digestive enzyme activity (57). *Faecalibacterium* was gram-negative, butyrate-producing bacteria, which was demonstrated to be negatively related to HbA1c (26). *Faecalibacterium* was proven to increase the intestinal synthesis of glucagon-like peptide-1, peptide YY, acetate and butyrate help to maintain glucose homeostasis (58). In this study, *Faecalibacterium* was depleted in DC, corroborating previous reports that linked *Faecalibacterium* to positive metabolic outcomes (29, 33, 59–61). These findings revealed that the alternation of gut microbiota at the genus level was closely related to DC, and the increase of *Bifidobacterium*, *Lactobacillus* and *Faecalibacterium* might be responsible for the pathology of DC. *Klebsiella* is a natural inhabitant of the gastrointestinal tract microbiome of healthy humans and animals, but it often causes extraintestinal infections, including urinary tract infections, pneumoniae and septicemia (62). Recently, *Klebsiella* was demonstrated to be increased in DKD (63), which was proven to be increased in DC when compared to HC in this study.

Microbiota symbiosis helped regulate metabolism and reduce the risk of DM. Gut microbiota dysbiosis was proven to lead imbalance of intestinal microbial bi-products, and cause insulin resistance, with mechanism unclear. Recently, the mechanism of how microbiota affect DM and its complications has raised academic interests. Firstly, microbiota dysbiosis led to the production of short-chain fatty acids (SCFAs) including butyrate, propionate, and acetate, which then impaired the intestinal barrier integrity, activated the inflammation signaling cascades and thus promoted the multi-organs damage (64). Secondly, microbiota-derived trimethylamine nitrogen oxide (TMAO) increased the accumulation of cholesterol, led to insulin resistance (65), and increased the risk of DM (66, 67). Third, changes in gut microbiota composition were proven to affect gut permeability and inflammation in DM (68, 69). Microbiota-derived lipopolysaccharide (LPS) triggered downstream inflammatory pathways and pro-inflammatory cytokine expression cascades, leading to inflammatory reactions and aggravating insulin resistance (70, 71). Last but not the least, microbiota-derived aromatic amino acids including indoxyl sulfate and p-cresyl sulfate, also known as uremic toxins, might induce mitochondrial dysfunction, podocytes injuries, thicken the glomerular basement membrane, and ultimately lead to renal micro-inflammation, and perivascular fibrosis in DKD (72, 73).

In this meta-analysis, genus *Bifidobacterium* belonged to the phylum Actinobacteria, genera *Faecalibacterium* and *Lactobacillus* belonged to the phylum Firmicutes, genus *Klebsiella* belonged to the phylum *Proteobacteria.* Phyla Actinobacteria, Firmicutes and Proteobacteria were both SCFAs-producing and LPS-producing bacteria. Therefore, we hypothesized that SCFAs-induced intestinal barrier integrity impairment and LPS-induced inflammation might be important parts of mechanism of DC. Nevertheless, direct targets of gut microbiota and the potential mechanisms needed to be further elucidated.

In spite of these interesting findings, our study was not without limitations. First, the language of included literature was limited to English, which may increase the possibility of language bias or publication bias. Secondly, since the gut microbiota was closely related to race and living environment, and the included studies originated mostly from Asia (11/13), in which nine studies were conducted in China and two in India. To explore the potential source of heterogeneity caused by it, the subgroup analysis on the nation has been performed in some comparison. The generalization of these results can only represent the characteristics of Asian populations to some extent. Third, in the 13 studies included, there were seven (7/13) studies focused on DKD, five (5/13) focused on DR, and only one (1/13) focused on DPN. To explore the potential source of heterogeneity caused by different microvascular complications of DM, the subgroup analysis on the type of microvascular complications has been performed. Considering the representativeness and potential bias of DPN, a meta-analysis of studies excluding DPN was performed to compare. Although DR and DKD were more representative microvascular complications in some ways, it would be better if a larger number of studies on DPN could be included in this meta-analysis. Fourth, the heterogeneity was high, although subgroup analysis on the type of DC, nation and city was performed, it was far from enough to explain the source of heterogeneity. More studies should be included and more factors related to microbiota such as ethnicity, eating habits, living environment, obesity condition, and even drugs on the changes of gut microbiota. Last, a different method of gene sequencing was also a potential bias on the results.

## Conclusions

In conclusion, we demonstrated that the alternation of alpha diversity, the abundances of phyla Proteobacteria and Firmicutes as well as the abundances of genera *Bifidobacterium*, *Faecalibacterium Lactobacillus* and *Klebsiella* may be associated with DC. The changes of microbial features may be noninvasive biomarkers for monitoring the management of DC. However, our findings still need to be verified by further large-scale, multi-center and high-quality studies. Nevertheless, conflicting results have also been reported, and therefore further studies would be necessary to fully understand the relation between gut microbiota and DC.

## Data availability statement

All data may be obtained within the article, the supplementary files, or from the original articles.

## Author contributions

JH, DL, and CM designed the study. JH and TF performed the literature search, screened for eligible studies, and extracted data, which were then analyzed by WL and YD. JH wrote the first draft of the manuscript and all authors contributed to subsequent drafts and approved the final version for submission.

## Funding

This study was funded by the Traditional Chinese Medicine Bureau of Guangdong Province (Nos. 20231003, 20211003 and 20223001), the National Natural Science Foundation of China (No. 82202560), the Natural Science Foundation of Guangdong Province (No. 2021A1515220050), the Fundamental Research Funds for the Central University (No. 22qntd4813) and the second batch of central subsidy funds for traditional Chinese medicine departments in 2022 (construction of national traditional Chinese medicine specialty) (No. 2022CZ0047).

## Acknowledgments

The authors acknowledged Guangdong Provincial People’s Hospital and Sun Yat-sen University for the academic supports.

## Conflict of interest

The authors declare that the research was conducted in the absence of any commercial or financial relationships that could be construed as a potential conflict of interest.

## Publisher’s note

All claims expressed in this article are solely those of the authors and do not necessarily represent those of their affiliated organizations, or those of the publisher, the editors and the reviewers. Any product that may be evaluated in this article, or claim that may be made by its manufacturer, is not guaranteed or endorsed by the publisher.

## References

[1] Khalili L Alipour B Asghari JM Hassanalilou T Mesgari AM Faraji I . Probiotic assisted weight management as a main factor for glycemic control in patients with type 2 diabetes: a randomized controlled trial. Diabetol Metab Syndr (2019) 11:5. doi: 10.1186/s13098-019-0400-7 30675190 PMC6334408

[2] Sivakumar PM Prabhawathi V Zarrabi A Akthar S Prabhakar PK . Current trends in the therapeutic strategies for diabetes management. Curr Med Chem (2021) 28:4616–37. doi: 10.2174/0929867328666210218183914 33602069

[3] Saeedi P Salpea P Karuranga S Petersohn I Malanda B Gregg EW . Mortality attributable to diabetes in 20-79 years old adults 2019 estimates: Results from the international diabetes federation diabetes atlas, 9(th) edition. Diabetes Res Clin Pract (2020) 162:108086. doi: 10.1016/j.diabres.2020.108086 32068099

[4] Cho NH Shaw JE Karuranga S Huang Y Da RFJ Ohlrogge AW . IDF diabetes atlas: Global estimates of diabetes prevalence for 2017 and projections for 2045. Diabetes Res Clin Pract (2018) 138:271–81. doi: 10.1016/j.diabres.2018.02.023 29496507

[5] Ahsan H . Diabetic retinopathy–biomolecules and multiple pathophysiology. Diabetes Metab Syndr (2015) 9:51–4. doi: 10.1016/j.dsx.2014.09.011 25450817

[6] Leasher JL Bourne RR Flaxman SR Jonas JB Keeffe J Naidoo K . Global estimates on the number of people blind or visually impaired by diabetic retinopathy: A meta-analysis from 1990 to 2010. Diabetes Care (2016) 39:1643–9. doi: 10.2337/dc15-2171 27555623

[7] Cui Y Zhang M Zhang L Zhang L Kuang J Zhang G . Prevalence and risk factors for diabetic retinopathy in a cross-sectional population-based study from rural southern China: Dongguan eye study. BMJ Open (2019) 9:e023586. doi: 10.1136/bmjopen-2018-023586 PMC6756414 31530585

[8] Javed S Hayat T Menon L Alam U Malik RA . Diabetic peripheral neuropathy in people with type 2 diabetes: too little too late. Diabetes Med (2020) 37:573–9. doi: 10.1111/dme.14194 31797434

[9] Iatcu CO Steen A Covasa M . Gut microbiota and complications of type-2 diabetes. Nutrients (2021) 14:166. doi: 10.3390/nu14010166 35011044 PMC8747253

[10] Fan Y Pedersen O . Gut microbiota in human metabolic health and disease. Nat Rev Microbiol (2021) 19:55–71. doi: 10.1038/s41579-020-0433-9 32887946

[11] Lin D Song Q Zhang Y Liu J Chen F Du S . Bacillus subtilis attenuates hepatic and intestinal injuries and modulates gut microbiota and gene expression profiles in mice infected with schistosoma japonicum. Front Cell Dev Biol (2021) 9:766205. doi: 10.3389/fcell.2021.766205 34869360 PMC8635066

[12] Dominguez-Bello MG Godoy-Vitorino F Knight R Blaser MJ . Role of the microbiome in human development. Gut (2019) 68:1108–14. doi: 10.1136/gutjnl-2018-317503 PMC6580755 30670574

[13] Lin D Song Q Liu J Chen F Zhang Y Wu Z . Potential gut microbiota features for non-invasive detection of schistosomiasis. Front Immunol (2022) 13:941530. doi: 10.3389/fimmu.2022.941530 35911697 PMC9330540

[14] Aron-Wisnewsky J Vigliotti C Witjes J Le P Holleboom AG Verheij J . Gut microbiota and human NAFLD: disentangling microbial signatures from metabolic disorders. Nat Rev Gastroenterol Hepatol (2020) 17:279–97. doi: 10.1038/s41575-020-0269-9 32152478

[15] Das T Jayasudha R Chakravarthy S Prashanthi GS Bhargava A Tyagi M . Alterations in the gut bacterial microbiome in people with type 2 diabetes mellitus and diabetic retinopathy. Sci Rep (2021) 11:2738. doi: 10.1038/s41598-021-82538-0 33531650 PMC7854632

[16] Wang Y Ye X Ding D Lu Y . Characteristics of the intestinal flora in patients with peripheral neuropathy associated with type 2 diabetes. J Int Med Res (2020) 48:300060520936806. doi: 10.1177/0300060520936806 32938282 PMC7503028

[17] He X Sun J Liu C Yu X Li H Zhang W . Compositional alterations of gut microbiota in patients with diabetic kidney disease and type 2 diabetes mellitus. Diabetes Metab Syndr Obes (2022) 15:755–65. doi: 10.2147/DMSO.S347805 PMC8911313 35280499

[18] Wu H Esteve E Tremaroli V Khan MT Caesar R Manneras-Holm L . Metformin alters the gut microbiome of individuals with treatment-naive type 2 diabetes, contributing to the therapeutic effects of the drug. Nat Med (2017) 23:850–8. doi: 10.1038/nm.4345 28530702

[19] Lee CB Chae SU Jo SJ Jerng UM Bae SK . The relationship between the gut microbiome and metformin as a key for treating type 2 diabetes mellitus. Int J Mol Sci (2021) 22:3566. doi: 10.3390/ijms22073566 33808194 PMC8037857

[20] Armour CR Nayfach S Pollard KS Sharpton TJ . A metagenomic meta-analysis reveals functional signatures of health and disease in the human gut microbiome. mSystems (2019) 4:e00332-18. doi: 10.1128/mSystems.00332-18 31098399 PMC6517693

[21] Hutton B Salanti G Caldwell DM Chaimani A Schmid CH Cameron C . The PRISMA extension statement for reporting of systematic reviews incorporating network meta-analyses of health care interventions: checklist and explanations. Ann Intern Med (2015) 162:777–84. doi: 10.7326/M14-2385 26030634

[22] Stang A . Critical evaluation of the Newcastle-Ottawa scale for the assessment of the quality of nonrandomized studies in meta-analyses. Eur J Epidemiol (2010) 25:603–5. doi: 10.1007/s10654-010-9491-z 20652370

[23] Nano J Muka T Cepeda M Voortman T Dhana K Brahimaj A . Association of circulating total bilirubin with the metabolic syndrome and type 2 diabetes: A systematic review and meta-analysis of observational evidence. Diabetes Metab (2016) 42:389–97. doi: 10.1016/j.diabet.2016.06.002 27396752

[24] Al-Obaide M Singh R Datta P Rewers-Felkins KA Salguero MV Al-Obaidi I . Gut microbiota-dependent trimethylamine-n-oxide and serum biomarkers in patients with T2DM and advanced CKD. J Clin Med (2017) 6:86. doi: 10.3390/jcm6090086 28925931 PMC5615279

[25] Salguero MV Al-Obaide M Singh R Siepmann T Vasylyeva TL . Dysbiosis of gram-negative gut microbiota and the associated serum lipopolysaccharide exacerbates inflammation in type 2 diabetic patients with chronic kidney disease. Exp Ther Med (2019) 18:3461–9. doi: 10.3892/etm.2019.7943 PMC6777309 31602221

[26] Tao S Li L Li L Liu Y Ren Q Shi M . Understanding the gut-kidney axis among biopsy-proven diabetic nephropathy, type 2 diabetes mellitus and healthy controls: an analysis of the gut microbiota composition. Acta Diabetol (2019) 56:581–92. doi: 10.1007/s00592-019-01316-7 30888537

[27] Jayasudha R Das T Kalyana CS Sai PG Bhargava A Tyagi M . Gut mycobiomes are altered in people with type 2 diabetes mellitus and diabetic retinopathy. PloS One (2020) 15:e0243077. doi: 10.1371/journal.pone.0243077 33259537 PMC7707496

[28] Du X Liu J Xue Y Kong X Lv C Li Z . Alteration of gut microbial profile in patients with diabetic nephropathy. Endocrine (2021) 73:71–84. doi: 10.1007/s12020-021-02721-1 33905112

[29] Huang Y Wang Z Ma H Ji S Chen Z Cui Z . Dysbiosis and implication of the gut microbiota in diabetic retinopathy. Front Cell Infect Microbiol (2021) 11:646348. doi: 10.3389/fcimb.2021.646348 33816351 PMC8017229

[30] Ye P Zhang X Xu Y Xu J Song X Yao K . Alterations of the gut microbiome and metabolome in patients with proliferative diabetic retinopathy. Front Microbiol (2021) 12:667632. doi: 10.3389/fmicb.2021.667632 34566901 PMC8457552

[31] Zhou Z Zheng Z Xiong X Chen X Peng J Yao H . Gut microbiota composition and fecal metabolic profiling in patients with diabetic retinopathy. Front Cell Dev Biol (2021) 9:732204. doi: 10.3389/fcell.2021.732204 34722512 PMC8554156

[32] Chen R Zhu D Yang R Wu Z Xu N Chen F . Gut microbiota diversity in middle-aged and elderly patients with end-stage diabetic kidney disease. Ann Transl Med (2022) 10:750. doi: 10.21037/atm-22-2926 35957707 PMC9358493

[33] Zhang L Wang Z Zhang X Zhao L Chu J Li H . Alterations of the gut microbiota in patients with diabetic nephropathy. Microbiol Spectr (2022) 10:e0032422. doi: 10.1128/spectrum.00324-22 35863004 PMC9430528

[34] Caudet J Trelis M Cifre S Soriano JM Rico H Merino-Torres JF . Interplay between intestinal bacterial communities and unicellular parasites in a morbidly obese population: A neglected trinomial. Nutrients (2022) 14:3211. doi: 10.3390/nu14153211 35956387 PMC9370494

[35] Zhang Y Lu S Yang Y Wang Z Wang B Zhang B . The diversity of gut microbiota in type 2 diabetes with or without cognitive impairment. Aging Clin Exp Res (2021) 33:589–601. doi: 10.1007/s40520-020-01553-9 32301029

[36] Du Y Neng Q Li Y Kang Y Guo L Huang X . Gastrointestinal autonomic neuropathy exacerbates gut microbiota dysbiosis in adult patients with type 2 diabetes mellitus. Front Cell Infect Microbiol (2021) 11:804733. doi: 10.3389/fcimb.2021.804733 35211420 PMC8861497

[37] Pussinen PJ Havulinna AS Lehto M Sundvall J Salomaa V . Endotoxemia is associated with an increased risk of incident diabetes. Diabetes Care (2011) 34:392–7. doi: 10.2337/dc10-1676 PMC3024355 21270197

[38] Prawitt J Caron S Staels B . Bile acid metabolism and the pathogenesis of type 2 diabetes. Curr Diabetes Rep (2011) 11:160–6. doi: 10.1007/s11892-011-0187-x PMC3338411 21431855

[39] Ley RE Turnbaugh PJ Klein S Gordon JI . Microbial ecology: human gut microbes associated with obesity. Nature (2006) 444:1022–3. doi: 10.1038/4441022a 17183309

[40] Krajmalnik-Brown R Ilhan ZE Kang DW DiBaise JK . Effects of gut microbes on nutrient absorption and energy regulation. Nutr Clin Pract (2012) 27:201–14. doi: 10.1177/0884533611436116 PMC3601187 22367888

[41] Kumar H Lund R Laiho A Lundelin K Ley RE Isolauri E . Gut microbiota as an epigenetic regulator: pilot study based on whole-genome methylation analysis. mBio (2014) 5:e02113-14. doi: 10.1128/mBio.02113-14 25516615 PMC4271550

[42] Welcome MO . Gut microbiota disorder, gut epithelial and blood-brain barrier dysfunctions in etiopathogenesis of dementia: Molecular mechanisms and signaling pathways. Neuromol Med (2019) 21:205–26. doi: 10.1007/s12017-019-08547-5 31115795

[43] Jumpertz R Le DS Turnbaugh PJ Trinidad C Bogardus C Gordon JI . Energy-balance studies reveal associations between gut microbes, caloric load, and nutrient absorption in humans. Am J Clin Nutr (2011) 94:58–65. doi: 10.3945/ajcn.110.010132 21543530 PMC3127503

[44] Patil DP Dhotre DP Chavan SG Sultan A Jain DS Lanjekar VB . Molecular analysis of gut microbiota in obesity among Indian individuals. J Biosci (2012) 37:647–57. doi: 10.1007/s12038-012-9244-0 22922190

[45] Tims S Derom C Jonkers DM Vlietinck R Saris WH Kleerebezem M . Microbiota conservation and BMI signatures in adult monozygotic twins. ISME J (2013) 7:707–17. doi: 10.1038/ismej.2012.146 PMC3603393 23190729

[46] Aguirre M Venema K . Does the gut microbiota contribute to obesity? going beyond the gut feeling. Microorganisms (2015) 3:213–35. doi: 10.3390/microorganisms3020213 PMC5023237 27682087

[47] Robles-Vera I Toral M de la Visitacion N Sanchez M Romero M Olivares M . The probiotic lactobacillus fermentum prevents dysbiosis and vascular oxidative stress in rats with hypertension induced by chronic nitric oxide blockade. Mol Nutr Food Res (2018) 62:e1800298. doi: 10.1002/mnfr.201800298 30028078

[48] Alessandri G van Sinderen D Ventura M . The genus bifidobacterium: From genomics to functionality of an important component of the mammalian gut microbiota running title: Bifidobacterial adaptation to and interaction with the host. Comput Struct Biotechnol J (2021) 19:1472–87. doi: 10.1016/j.csbj.2021.03.006 PMC7979991 33777340

[49] Moroti C Souza ML de Rezende CM Cavallini DC Sivieri K . Effect of the consumption of a new symbiotic shake on glycemia and cholesterol levels in elderly people with type 2 diabetes mellitus. Lipids Health Dis (2012) 11:29. doi: 10.1186/1476-511X-11-29 22356933 PMC3305430

[50] Cani PD . Microbiota and metabolites in metabolic diseases. Nat Rev Endocrinol (2019) 15:69–70. doi: 10.1038/s41574-018-0143-9 30602737

[51] Zou Y Chen T . Engineered akkermansia muciniphila: A promising agent against diseases (Review). Exp Ther Med (2020) 20:285. doi: 10.3892/etm.2020.9415 33209129 PMC7668130

[52] Riviere A Selak M Lantin D Leroy F De Vuyst L . Bifidobacteria and butyrate-producing colon bacteria: Importance and strategies for their stimulation in the human gut. Front Microbiol (2016) 7:979. doi: 10.3389/fmicb.2016.00979 27446020 PMC4923077

[53] Wu X Ma C Han L Nawaz M Gao F Zhang X . Molecular characterisation of the faecal microbiota in patients with type II diabetes. Curr Microbiol (2010) 61:69–78. doi: 10.1007/s00284-010-9582-9 20087741

[54] Sroka-Oleksiak A Mlodzinska A Bulanda M Salamon D Major P Stanek M . Metagenomic analysis of duodenal microbiota reveals a potential biomarker of dysbiosis in the course of obesity and type 2 diabetes: A pilot study. J Clin Med (2020) 9:369. doi: 10.3390/jcm9020369 32013181 PMC7074165

[55] Ejtahed HS Mohtadi-Nia J Homayouni-Rad A Niafar M Asghari-Jafarabadi M Mofid V . Probiotic yogurt improves antioxidant status in type 2 diabetic patients. Nutrition (2012) 28:539–43. doi: 10.1016/j.nut.2011.08.013 22129852

[56] Ostadrahimi A Taghizadeh A Mobasseri M Farrin N Payahoo L Beyramalipoor GZ . Effect of probiotic fermented milk (kefir) on glycemic control and lipid profile in type 2 diabetic patients: a randomized double-blind placebo-controlled clinical trial. Iran J Public Health (2015) 44:228–37. PMC4401881 25905057

[57] Dowarah R Verma AK Agarwal N . The use of lactobacillus as an alternative of antibiotic growth promoters in pigs: A review. Anim Nutr (2017) 3:1–6. doi: 10.1016/j.aninu.2016.11.002 29767055 PMC5941084

[58] Huda MN Kim M Bennett BJ . Modulating the microbiota as a therapeutic intervention for type 2 diabetes. Front Endocrinol (Lausanne) (2021) 12:632335. doi: 10.3389/fendo.2021.632335 33897618 PMC8060771

[59] Sokol H Pigneur B Watterlot L Lakhdari O Bermudez-Humaran LG Gratadoux JJ . Faecalibacterium prausnitzii is an anti-inflammatory commensal bacterium identified by gut microbiota analysis of crohn disease patients. Proc Natl Acad Sci U.S.A. (2008) 105:16731–6. doi: 10.1073/pnas.0804812105 PMC2575488 18936492

[60] Le Chatelier E Nielsen T Qin J Prifti E Hildebrand F Falony G . Richness of human gut microbiome correlates with metabolic markers. Nature (2013) 500:541–6. doi: 10.1038/nature12506 23985870

[61] Thingholm LB Ruhlemann MC Koch M Fuqua B Laucke G Boehm R . Obese individuals with and without type 2 diabetes show different gut microbial functional capacity and composition. Cell Host Microbe (2019) 26:252–264.e10. doi: 10.1016/j.chom.2019.07.004 31399369 PMC7720933

[62] Navon-Venezia S Kondratyeva K Carattoli A . Klebsiella pneumoniae: a major worldwide source and shuttle for antibiotic resistance. FEMS Microbiol Rev (2017) 41:252–75. doi: 10.1093/femsre/fux013 28521338

[63] Wang Y Zhao J Qin Y Yu Z Zhang Y Ning X . The specific alteration of gut microbiota in diabetic kidney diseases-a systematic review and meta-analysis. Front Immunol (2022) 13:908219. doi: 10.3389/fimmu.2022.908219 35784273 PMC9248803

[64] Schippa S Conte MP . Dysbiotic events in gut microbiota: impact on human health. Nutrients (2014) 6:5786–805. doi: 10.3390/nu6125786 PMC4276999 25514560

[65] Oellgaard J Winther SA Hansen TS Rossing P von Scholten BJ . Trimethylamine n-oxide (TMAO) as a new potential therapeutic target for insulin resistance and cancer. Curr Pharm Des (2017) 23:3699–712. doi: 10.2174/1381612823666170622095324 28641532

[66] Fernandes J Su W Rahat-Rozenbloom S Wolever TM Comelli EM . Adiposity, gut microbiota and faecal short chain fatty acids are linked in adult humans. Nutr Diabetes (2014) 4:e121. doi: 10.1038/nutd.2014.23 24979150 PMC4079931

[67] Shan Z Sun T Huang H Chen S Chen L Luo C . Association between microbiota-dependent metabolite trimethylamine-n-oxide and type 2 diabetes. Am J Clin Nutr (2017) 106:888–94. doi: 10.3945/ajcn.117.157107 28724646

[68] Cani PD Possemiers S Van de Wiele T Guiot Y Everard A Rottier O . Changes in gut microbiota control inflammation in obese mice through a mechanism involving GLP-2-driven improvement of gut permeability. Gut (2009) 58:1091–103. doi: 10.1136/gut.2008.165886 PMC2702831 19240062

[69] Boutagy NE McMillan RP Frisard MI Hulver MW . Metabolic endotoxemia with obesity: Is it real and is it relevant? Biochimie (2016) 124:11–20. doi: 10.1016/j.biochi.2015.06.020 26133659 PMC4695328

[70] Zanoni I Ostuni R Marek LR Barresi S Barbalat R Barton GM . CD14 controls the LPS-induced endocytosis of toll-like receptor 4. Cell (2011) 147:868–80. doi: 10.1016/j.cell.2011.09.051 PMC3217211 22078883

[71] Wen L Duffy A . Factors influencing the gut microbiota, inflammation, and type 2 diabetes. J Nutr (2017) 147:1468S–75S. doi: 10.3945/jn.116.240754 PMC5483960 28615382

[72] Hsiao EY McBride SW Hsien S Sharon G Hyde ER McCue T . Microbiota modulate behavioral and physiological abnormalities associated with neurodevelopmental disorders. Cell (2013) 155:1451–63. doi: 10.1016/j.cell.2013.11.024 PMC3897394 24315484

[73] Kikuchi K Saigusa D Kanemitsu Y Matsumoto Y Thanai P Suzuki N . Gut microbiome-derived phenyl sulfate contributes to albuminuria in diabetic kidney disease. Nat Commun (2019) 10:1835. doi: 10.1038/s41467-019-09735-4 31015435 PMC6478834

