# Supplementary material for: Specific alterations of gut microbiota in diabetic microvascular complications: A systematic review and meta-analysis
Source: Front Endocrinol (Lausanne). 2022 Dec 5;13:1053900. doi: 10.3389/fendo.2022.1053900 (PMC9761769; doi:10.3389/fendo.2022.1053900)
Supplement: Supplementary file 1 [file DataSheet_1.docx]

**Figure legends of supplementary tables and figures**

Supplementary Table S1: Newcastle-Ottawa Quality Assessment Scale of studies included.

Supplementary FIGURE S1: Bias assessment of alpha diversity in DC v.s. DM. (A) Forest plot of OTUs, within China and out of China; (B) Forest plot of OTUs, with different city; (C) Forest plot of Chao1, within China and out of China; (D) Forest plot of Shannon, within China and out of China; (E) Funnel plot of OTUs. (F) Funnel plot of Chao1; (G) Funnel plot of Shannon.

Supplementary FIGURE S2: Forest plots of alpha diversity in DC v.s. DM. (A) Simpson; (B) Shannon; (C) ACE; (D) Chao1.

Supplementary FIGURE S3: Bias assessment of Shannon and Chao1 in DC v.s. DM. (A) Forest plot of Shannon, within China and out of China; (B) Forest plot of Chao1, within China and out of China; (C) Funnel plot of Shannon; (D) Funnel plot of Chao1.

Supplementary FIGURE S4: Bias assessment of OTUs in DC v.s. HC. (A) Forest plot within China and out of China; (B) Funnel plot.

Supplementary FIGURE S5: Bias assessment of alpha diversity in DC v.s. HC. (A) Forest plot of Chao1 within China and out of China; (B) Forest plot of Shannon within China and out of China; (C) Funnel plot of Chao1; (D) Funnel plot of Shannon; (E) Funnel plot of ACE.

Supplementary FIGURE S6: Forest plots of alpha diversity in DC v.s. HC. (A) ACE; (B) Chao1; (C) Simpson; (D) Shannon.

Supplementary FIGURE S7: Bias assessment of alpha diversity in DC v.s. HC. (A) Forest plot of Chao1 within China and out of China; (B) Forest plot of Shannon within China and out of China; (C) Funnel plot of Chao1; (D) Funnel plot of Shannon; (E) Funnel plot of ACE.

Supplementary FIGURE S8: Forest plots of microbiota at the phylum level in DC v.s. DM. (A) Fusobacteria; (B) Verrucomicrobia.

Supplementary FIGURE S9: Forest plots of microbiota at the phylum level in DC v.s. HC. (A) Actinobacteria; (B)Verrucomicrobia.

Supplementary FIGURE S10: Forest plots of microbiota at the genus level in DC v.s. DM. (A) *Alistipes*; (B) *Prevotella*; (C) *Ruminococcus*; (D) *Lachnospira*; (E) *Roseburia*; (F) *Clostridium*.

Supplementary FIGURE S11: Forest plots of microbiota at the genus level in DC v.s. DM. (A) *Blautia*; (B) *Escherichia*; (C) *Eubacterium*; (D) *Parabacteroides*; (E) *Mitsuokella*; (F) *Lactobacillus*.

Supplementary FIGURE S12: Forest plots of microbiota at the genus level in DC v.s. HC. (A) *Streptococcus*; (B) *Roseburia*; (C) *Clostridium*; (D) *Blautia*.

Supplementary FIGURE S13: Forest plots of microbiota at the genus level in DC v.s. HC. (A) *Escherichia*; (B) *Eubacterium*; (C) *Bifidobacterium*; (D) *Lachnospira*.
